# Supplementary material for: End Sequence Analysis Toolkit (ESAT) expands the extractable information from single-cell RNA-seq data
Source: Genome Res. 2016 Oct;26(10):1397–410. doi: 10.1101/gr.207902.116 (PMC5052061; doi:10.1101/gr.207902.116)
Supplement: Supplemental Material [file supp_gr.207902.116_Supp_Materials.tar.gz › ESAT-esat_v0.1/doc/html/classbroad_1_1pda_1_1seq_1_1graph_1_1_path.html]

ESAT: broad.pda.seq.graph.Path Class Reference


ESAT


- Main Page
- Related Pages
- Packages
- Classes
- Files

- Class List
- Class Index
- Class Hierarchy
- Class Members

All Classes Namespaces Files Functions Variables Enumerator Pages

- broad
- pda
- seq
- graph
- Path

Classes |
Public Member Functions |
List of all members

broad.pda.seq.graph.Path Class Reference

Inheritance diagram for broad.pda.seq.graph.Path:

[legend]

Collaboration diagram for broad.pda.seq.graph.Path:

[legend]

|  |  |
| --- | --- |
| Classes | |
| class | **StandardTransformer** |
|  | |

|  |  |
| --- | --- |
| Public Member Functions | |
|  | Path (Collection< BubbleEdge > edges, double spliceWeight) |
|  | |
|  | Path (Collection< BubbleEdge > edges, Transformer< BubbleEdge, Number > transformer, double spliceWeight) |
|  | |
|  | Path (double spliceWeight) |
|  | |
|  | Path () |
|  | |
|  | Path (Gene gene, ChromosomeWithBubblesJGraphT graph) |
|  | |
| boolean | addPairedEnd (Alignment align) |
|  | |
| double | getScore () |
|  | |
| double | getWeight () |
|  | |
| int | getStart () |
|  | |
| int | getEnd () |
|  | |
| String | getChromosome () |
|  | |
| boolean | addEdge (BubbleEdge edge) |
|  | |
| boolean | addEdges (Collection< BubbleEdge > edges) |
|  | |
| Collection< BubbleEdge > | getEdges () |
|  | |
| List< BubbleEdge > | getEdgeList () |
|  | |
| boolean | removeEdge (BubbleEdge edge) |
|  | |
| int | getNumberOfEdges () |
|  | |
| Gene | toGene () |
|  | |
| Gene | toGene (double[] extraFields) |
|  | |
| String | getOrientation () |
|  | |
| int | compareTo (Path b) |
|  | |
| boolean | equals (Object o) |
|  | |
| int | hashCode () |
|  | |
| Alignments | getAlignment () |
|  | |
| Collection< Alignment > | getPairedEndEdges () |
|  | |
| void | addPairedEndEdges (Collection< Alignment > pairedEndEdges2) |
|  | |
| String | toString () |
|  | |
| Collection< Annotation > | getSortedNodes () |
|  | |
| Annotation | getStartVertex () |
|  | |
| Annotation | getEndVertex () |
|  | |
| boolean | overlapsLeft (Alignment read) |
|  | |
| Collection< Annotation > | getOverlappingNodes (Alignment read) |
|  | |
| boolean | overlapsRight (Alignment read) |
|  | |
| boolean | overlaps (Path leftPath) |
|  | |
| double | getPercentConsistent (EmpiricalDistribution pairedInsertDistribution) |
|  | |
| Path | copy () |
|  | |
| double | getCoverage () |
|  | |
| int | getSize () |
|  | |
| void | setLocalLambda (double localLambda) |
|  | |
| double | getLocalLambda () |
|  | |
| Gene | toFilledInGene () |
|  | |
| double | getNodeCount (Annotation v1) |
|  | |
| void | setSpliceWeight (double weight) |
|  | |
| double | getSpliceWeight () |
|  | |
| void | setIsToComplex (boolean isToComplex) |
|  | |
| boolean | isToComplex () |
|  | |
| Graph< Annotation, BubbleEdge > | getGraph () |
|  | |

## Constructor & Destructor Documentation

|  |  |  |  |
| --- | --- | --- | --- |
| broad.pda.seq.graph.Path.Path | ( | Collection< BubbleEdge > | *edges*, |
|  |  | double | *spliceWeight* |
|  | ) |  |  |

|  |  |  |  |
| --- | --- | --- | --- |
| broad.pda.seq.graph.Path.Path | ( | Collection< BubbleEdge > | *edges*, |
|  |  | Transformer< BubbleEdge, Number > | *transformer*, |
|  |  | double | *spliceWeight* |
|  | ) |  |  |

|  |  |  |  |  |  |
| --- | --- | --- | --- | --- | --- |
| broad.pda.seq.graph.Path.Path | ( | double | *spliceWeight* | ) |  |

|  |  |  |  |  |
| --- | --- | --- | --- | --- |
| broad.pda.seq.graph.Path.Path | ( |  | ) |  |

Here is the caller graph for this function:

|  |  |  |  |
| --- | --- | --- | --- |
| broad.pda.seq.graph.Path.Path | ( | Gene | *gene*, |
|  |  | ChromosomeWithBubblesJGraphT | *graph* |
|  | ) |  |  |

## Member Function Documentation

|  |  |  |  |  |  |
| --- | --- | --- | --- | --- | --- |
| boolean broad.pda.seq.graph.Path.addEdge | ( | BubbleEdge | *edge* | ) |  |

Here is the caller graph for this function:

|  |  |  |  |  |  |
| --- | --- | --- | --- | --- | --- |
| boolean broad.pda.seq.graph.Path.addEdges | ( | Collection< BubbleEdge > | *edges* | ) |  |

Here is the call graph for this function:

|  |  |  |  |  |  |
| --- | --- | --- | --- | --- | --- |
| boolean broad.pda.seq.graph.Path.addPairedEnd | ( | Alignment | *align* | ) |  |

Here is the call graph for this function:

|  |  |  |  |  |  |
| --- | --- | --- | --- | --- | --- |
| void broad.pda.seq.graph.Path.addPairedEndEdges | ( | Collection< Alignment > | *pairedEndEdges2* | ) |  |

|  |  |  |  |  |  |
| --- | --- | --- | --- | --- | --- |
| int broad.pda.seq.graph.Path.compareTo | ( | Path | *b* | ) |  |

Here is the call graph for this function:

|  |  |  |  |  |
| --- | --- | --- | --- | --- |
| Path broad.pda.seq.graph.Path.copy | ( |  | ) |  |

Here is the call graph for this function:

|  |  |  |  |  |  |
| --- | --- | --- | --- | --- | --- |
| boolean broad.pda.seq.graph.Path.equals | ( | Object | *o* | ) |  |

Here is the call graph for this function:

|  |  |  |  |  |
| --- | --- | --- | --- | --- |
| Alignments broad.pda.seq.graph.Path.getAlignment | ( |  | ) |  |

Here is the call graph for this function:

|  |  |  |  |  |
| --- | --- | --- | --- | --- |
| String broad.pda.seq.graph.Path.getChromosome | ( |  | ) |  |

Here is the caller graph for this function:

|  |  |  |  |  |
| --- | --- | --- | --- | --- |
| double broad.pda.seq.graph.Path.getCoverage | ( |  | ) |  |

|  |  |  |  |  |
| --- | --- | --- | --- | --- |
| List<BubbleEdge> broad.pda.seq.graph.Path.getEdgeList | ( |  | ) |  |

Returns the edges making up the path. The first edge in this path is incident to the start vertex. The last edge is incident to the end vertex. The vertices along the path can be obtained by traversing from the start vertex, finding its opposite across the first edge, and then doing the same successively across subsequent edges; Graphs#getPathVertexList provides a convenience method for this.

Whether or not the returned edge list is modifiable depends on the path implementation.

Returns
:   list of edges traversed by the path

|  |  |  |  |  |
| --- | --- | --- | --- | --- |
| Collection<BubbleEdge> broad.pda.seq.graph.Path.getEdges | ( |  | ) |  |

|  |  |  |  |  |
| --- | --- | --- | --- | --- |
| int broad.pda.seq.graph.Path.getEnd | ( |  | ) |  |

TODO: This may be to slow, maybe compute end on the fly as Path is being constructed

Returns
:   genomic end of the path.

Here is the caller graph for this function:

|  |  |  |  |  |
| --- | --- | --- | --- | --- |
| Annotation broad.pda.seq.graph.Path.getEndVertex | ( |  | ) |  |

Returns the end vertex in the path.

Returns
:   the end vertex

Here is the call graph for this function:

|  |  |  |  |  |
| --- | --- | --- | --- | --- |
| Graph<Annotation, BubbleEdge> broad.pda.seq.graph.Path.getGraph | ( |  | ) |  |

Returns the graph over which this path is defined. The path may also be valid with respect to other graphs.

Returns
:   the containing graph

|  |  |  |  |  |
| --- | --- | --- | --- | --- |
| double broad.pda.seq.graph.Path.getLocalLambda | ( |  | ) |  |

|  |  |  |  |  |  |
| --- | --- | --- | --- | --- | --- |
| double broad.pda.seq.graph.Path.getNodeCount | ( | Annotation | *v1* | ) |  |

|  |  |  |  |  |
| --- | --- | --- | --- | --- |
| int broad.pda.seq.graph.Path.getNumberOfEdges | ( |  | ) |  |

|  |  |  |  |  |
| --- | --- | --- | --- | --- |
| String broad.pda.seq.graph.Path.getOrientation | ( |  | ) |  |

Here is the caller graph for this function:

|  |  |  |  |  |  |
| --- | --- | --- | --- | --- | --- |
| Collection<Annotation> broad.pda.seq.graph.Path.getOverlappingNodes | ( | Alignment | *read* | ) |  |

|  |  |  |  |  |
| --- | --- | --- | --- | --- |
| Collection<Alignment> broad.pda.seq.graph.Path.getPairedEndEdges | ( |  | ) |  |

|  |  |  |  |  |  |
| --- | --- | --- | --- | --- | --- |
| double broad.pda.seq.graph.Path.getPercentConsistent | ( | EmpiricalDistribution | *pairedInsertDistribution* | ) |  |

|  |  |  |  |  |
| --- | --- | --- | --- | --- |
| double broad.pda.seq.graph.Path.getScore | ( |  | ) |  |

Here is the caller graph for this function:

|  |  |  |  |  |
| --- | --- | --- | --- | --- |
| int broad.pda.seq.graph.Path.getSize | ( |  | ) |  |

|  |  |  |  |  |
| --- | --- | --- | --- | --- |
| Collection<Annotation> broad.pda.seq.graph.Path.getSortedNodes | ( |  | ) |  |

Here is the caller graph for this function:

|  |  |  |  |  |
| --- | --- | --- | --- | --- |
| double broad.pda.seq.graph.Path.getSpliceWeight | ( |  | ) |  |

|  |  |  |  |  |
| --- | --- | --- | --- | --- |
| int broad.pda.seq.graph.Path.getStart | ( |  | ) |  |

TODO: This may be to slow, maybe compute start on the fly as Path is being constructed

Returns
:   genomic start of the path.

Here is the caller graph for this function:

|  |  |  |  |  |
| --- | --- | --- | --- | --- |
| Annotation broad.pda.seq.graph.Path.getStartVertex | ( |  | ) |  |

Returns the start vertex in the path.

Returns
:   the start vertex

Here is the call graph for this function:

|  |  |  |  |  |
| --- | --- | --- | --- | --- |
| double broad.pda.seq.graph.Path.getWeight | ( |  | ) |  |

Returns the weight assigned to the path. Typically, this will be the sum of the weights of the edge list entries (as defined by the containing graph), but some path implementations may use other definitions.

Returns
:   the weight of the path

|  |  |  |  |  |
| --- | --- | --- | --- | --- |
| int broad.pda.seq.graph.Path.hashCode | ( |  | ) |  |

Here is the call graph for this function:

|  |  |  |  |  |
| --- | --- | --- | --- | --- |
| boolean broad.pda.seq.graph.Path.isToComplex | ( |  | ) |  |

Here is the call graph for this function:

Here is the caller graph for this function:

|  |  |  |  |  |  |
| --- | --- | --- | --- | --- | --- |
| boolean broad.pda.seq.graph.Path.overlaps | ( | Path | *leftPath* | ) |  |

Here is the call graph for this function:

|  |  |  |  |  |  |
| --- | --- | --- | --- | --- | --- |
| boolean broad.pda.seq.graph.Path.overlapsLeft | ( | Alignment | *read* | ) |  |

Here is the caller graph for this function:

|  |  |  |  |  |  |
| --- | --- | --- | --- | --- | --- |
| boolean broad.pda.seq.graph.Path.overlapsRight | ( | Alignment | *read* | ) |  |

|  |  |  |  |  |  |
| --- | --- | --- | --- | --- | --- |
| boolean broad.pda.seq.graph.Path.removeEdge | ( | BubbleEdge | *edge* | ) |  |

|  |  |  |  |  |  |
| --- | --- | --- | --- | --- | --- |
| void broad.pda.seq.graph.Path.setIsToComplex | ( | boolean | *isToComplex* | ) |  |

Here is the call graph for this function:

|  |  |  |  |  |  |
| --- | --- | --- | --- | --- | --- |
| void broad.pda.seq.graph.Path.setLocalLambda | ( | double | *localLambda* | ) |  |

|  |  |  |  |  |  |
| --- | --- | --- | --- | --- | --- |
| void broad.pda.seq.graph.Path.setSpliceWeight | ( | double | *weight* | ) |  |

|  |  |  |  |  |
| --- | --- | --- | --- | --- |
| Gene broad.pda.seq.graph.Path.toFilledInGene | ( |  | ) |  |

Here is the call graph for this function:

|  |  |  |  |  |
| --- | --- | --- | --- | --- |
| Gene broad.pda.seq.graph.Path.toGene | ( |  | ) |  |

Here is the call graph for this function:

Here is the caller graph for this function:

|  |  |  |  |  |  |
| --- | --- | --- | --- | --- | --- |
| Gene broad.pda.seq.graph.Path.toGene | ( | double[] | *extraFields* | ) |  |

Here is the call graph for this function:

|  |  |  |  |  |
| --- | --- | --- | --- | --- |
| String broad.pda.seq.graph.Path.toString | ( |  | ) |  |

Here is the call graph for this function:

---

The documentation for this class was generated from the following file:

- java/broad/pda/seq/graph/Path.java


---

Generated on Fri Aug 15 2014 15:11:02 for ESAT by  

 1.8.7
